# Quantitative analysis of motor evoked potentials in the neonatal lamb

**DOI:** 10.1038/s41598-017-16453-8

**Published:** 2017-11-23

**Authors:** Luc Joyeux, Marjolijn Deprez, Ahmad Khatoun, Kris Van Kuyck, Kelly Pelsmaekers, Alexander C. Engels, Hongmei Wang, Marina Gabriela Monteiro Carvalho Mori da Cunha, Stephanie De Vleeschauwer, Myles Mc Laughlin, Jan Deprest

**Affiliations:** 1 Academic Department Development and Regeneration, Cluster Organ Systems, Biomedical Sciences, Faculty of Medicine, Katholieke Universiteit (KU) Leuven, Leuven, Belgium; 2 Center for Surgical Technologies, Faculty of Medicine, KU Leuven, Leuven, Belgium; 3 Research group Experimental Neurosurgery and Neuroanatomy, Department of Neurosciences, KU Leuven, Leuven, Belgium; 4 Experimental Otorhinolaryngology, Department of Neurosciences, KU Leuven, Belgium; 5 Department of Obstetrics and Gynecology, Division Woman and Child, Fetal Medicine Unit, University Hospital Gasthuisberg UZ Leuven, Leuven, Belgium; 6 Department of Obstetrics and Gynecology, Shandong Provincial University Hospital, Jinan, China; 7 Animal Research Center, Biomedical Sciences, KU Leuven, Leuven, Belgium; 8 Institute of Women’s Health, University College London Hospitals, London, United Kingdom

## Abstract

Evoking motor potentials are an objective assessment method for neuromotor function, yet this was to our knowledge never done in neonatal lambs. There is neither a method for standardized quantification of motor evoked potentials (MEPs). We first aimed to evaluate the feasibility of MEP recording in neonatal lambs and test its validity. Second we aimed to develop an algorithm for its quantification and test its reliability since manual input is required. We recorded myogenic MEPs after transcranial motor cortex stimulation in 6 lambs aged 1–2 days. MEPs were also measured in one lamb undergoing Neuro-Muscular Blockade (NMB) and another undergoing lumbar spinal cord (SC) transection, both serving as controls. We computed 5 parameters using a custom-made algorithm: motor threshold, latency, area-under-the-curve, peak-to-peak amplitude and duration. Intra- and inter-observer reliability was analyzed. MEPs could be easily recorded, disappearing after NMB and SC transection. The algorithm allowed for analysis, hence physiologic readings of the parameters in all 4 limbs of all lambs were obtained. Our method was shown to have high intra- and inter-observer ( ≥70%) reliability for latency, area-under-the-curve and peak-to-peak amplitude. These results suggest that standardized MEP recording and analysis in neonatal lambs is feasible, and can reliably assess neuromotor function.

## Introduction

Motor evoked potentials (MEPs) are neuroelectrical signals produced by the spinal cord or peripheral muscles under transcranial or direct brain stimulation. MEPs provide direct and objective *in vivo* assessment of the function of involved central motor pathways, i.e. pyramidal tracts^1,2^. The neural response triggered by electrical or magnetic stimulation of the motor cortex crosses the midline in the brainstem, travels via the corticospinal motor pathways in the upper and lower motor neurons and ends in the muscle generating a muscle contraction^2,3^. Compared to sensory pathways, motor pathways are more sensitive to ischemic insults, and therefore MEPs have a better correlation with good motor outcome than somatosensory evoked potentials (SEPs)^4^. Clinically, MEPs are mainly indicated for routine intraoperative neuromonitoring (IONM) to assess motor functional integrity during surgeries associated with high risks of motor injury. They are indeed a recent addition to IONM during surgeries for brain, brainstem, spinal cord or peripheral nerve tumors or lesions^5^. Moreover some groups use MEPs for research purposes to study the pathophysiology of multiple sclerosis or motor neuron disease, and as prognostic indicators for response to treatment or for motor recovery in stroke, Parkinson’s disease, epilepsy and spina bifida (SB)^2,5–10^.

Experimentally, MEPs are studied in different animal models of brain, spinal cord, motor neuron and nerve diseases to assess motor functional integrity^6,11–15^. The neonatal lamb model is increasingly used in research to evaluate new fetal and neonatal therapies, such as fetal surgery for SB and cardiac congenital abnormalities or the artificial placenta^16–20^. To the best of our knowledge there is currently no objective way to assess the neuromotor function and MEPs have never been recorded in this model. In fact neither neurological and behavioral examinations, brain or spinal cord imaging nor SEPs can provide objective functional information about preservation of motor function. SEPs were solely developed in neonatal lambs to objectively and specifically evaluate sensory function^21^.

Variability, anesthetic vulnerability, fade and high sensitivity make MEP analysis, especially during or after IONM, more difficult and controversial. Currently, there is no consensus as to what constitutes an appropriate alarm criterion for myogenic MEP monitoring during IONM^5^. MEP interpretation is indeed based on binary and semi-quantitative alarm criteria like MEP disappearance, amplitude reduction, acute threshold elevation or morphology simplification^2,5,22^. To the best of our knowledge, a method for standardized quantification of MEPs does not exist yet.

Herein we aimed to evaluate the feasibility of MEP recording in neonatal lambs and test its validity. For the latter, we planned pharmacologic and surgical Neuro-Muscular Blockade (NMB) in order to confirm the presence of genuine MEPs. In addition, we aimed to develop an algorithm for MEP quantification and test its reliability since manual input is still required.

## Methods

This experiment was approved by the KU Leuven Ethical Committee on Animal Experimentation (# P285-2014). Our study followed the NC3Rs (National Center for the Replacement, Refinement, and Reduction of Animals in Research) and the ARRIVE (Animals in Research: Reporting *In Vivo* Experiments) guidelines for animal research^23,24^.

As we aimed to test the feasibility of MEP recording, the sample size calculation could not be based on power analysis^25,26^. We first conducted a pilot study in three neonatal lambs to develop a standardized MEP protocol. Then, using this standardized protocol, we conducted this feasibility study in six normal neonatal lambs. We added two extra lambs for the validity analysis. The lambs were chronologically allocated to the study.

### Surgical procedures

Eight time-dated pregnant sheep (Swifter breed) were provided by the KU Leuven Zoötechnisch Centrum (Lovenjoel, Belgium). One lamb per ewe was delivered by cesarean section through flank incision around term (mean delivery date, 144 ± 0.8 days of gestation; term, 145 days) under spinal anesthesia (Lignocaine hydrochloride 2%-adrenaline, 100 mL, Kela N.V., Hoogstraten, Belgium). After delivery, the lambs were nursed and given colostrum and lamb artificial milk (Ovilac 228/8, Aveve, Leuven Belgium) ad libitum in the first eight hours, and thereafter four-hourly. Neonatal lambs were assessed within 48 hours postnatally by a board-certified veterinarian using a standardized neurological clinical examination protocol for large animals^27^.

Right after feeding the lamb with milk, general anesthesia was induced with intravenous propofol (Propovet Multidose 10 mg/mL, Abbot, Breda, the Nederlands) administered through a 20-Ga cannula in the jugular vein^1,2^. An initial bolus of 5 mg/kg was given, with additional boluses of half this dose as necessary to abolish palpebral reflexes. At the same time, systemic hydration was maintained with boluses of 2 mL of a mixture of 2/3 of Hartmann’s solution (Hartmann® 1000 mL, Baxter Healthcare, Braine-l’Alleud, Belgium) and 1/3 of glucose 20% (Glucose 20%® 500 mL, Baxter Healthcare) to keep the animal hemodynamically stable. The lamb was kept warm at basal body temperature (39.4 ± 0.6 °C; rectal thermometer) before and between recordings using a heating pad^28^. All animals were euthanized with an overdose of propofol at the conclusion of the experiment.

**Figure 1:**
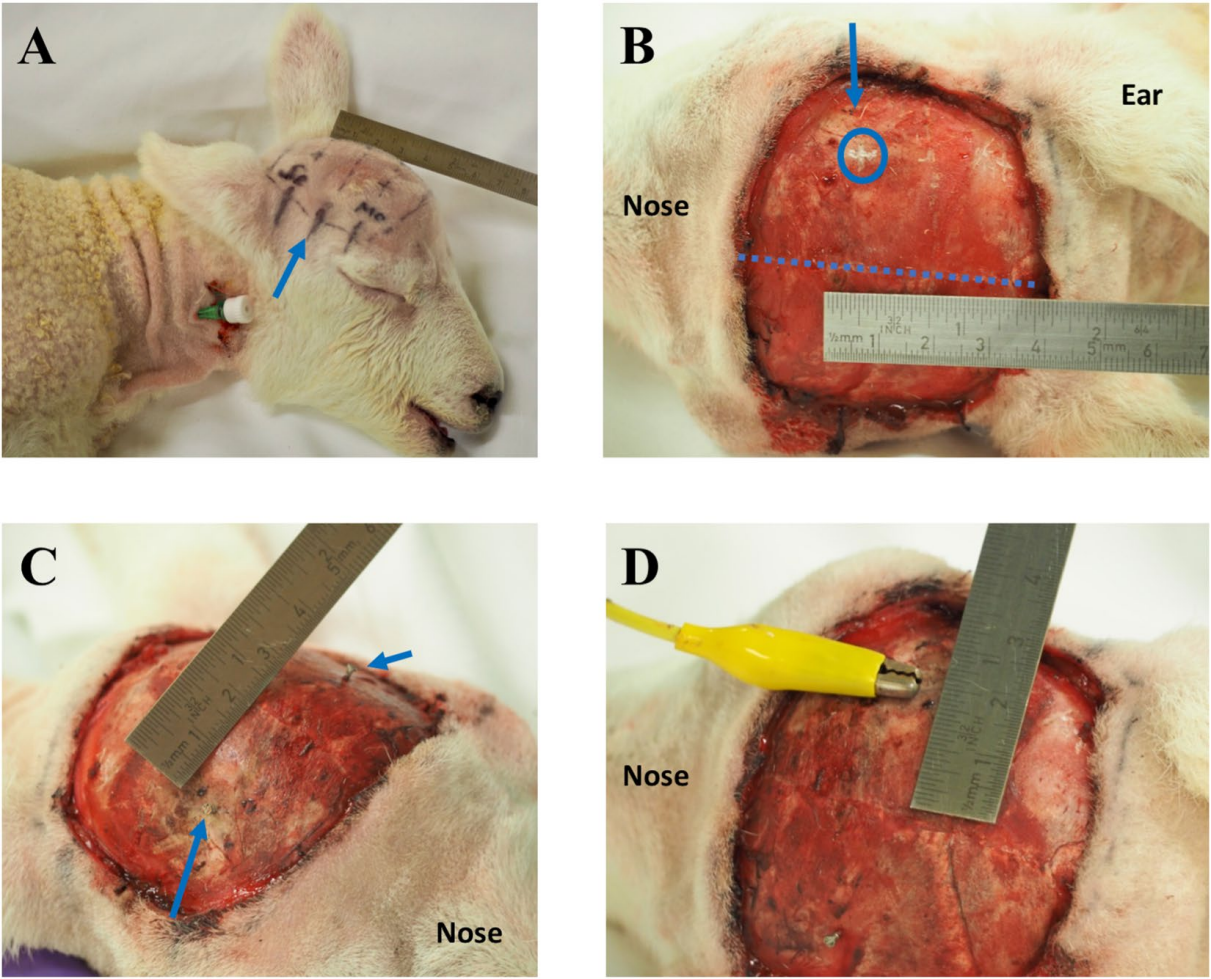
Transcranial electrode implantations. (**A**) External skin landmarks of the motor cortex (Mo), the cruciate sulcus (blue arrow) and the somatosensory cortex (Se), which are respectively located from 1 mm to 3 cm lateral to the midline (ruler) at the level of the posterior margin of the eyelid, from 0 to 4.5 cm lateral to the midline at the level of the posterior margin of the orbit and from 1 mm to 3 cm lateral to the midline at the level of the anterior edge of the ear. (**B)** Skull landmarks of the skull-screw electrode (encircled cross) located over the hind- and forelimb motor cortical representations, 2 cm lateral to the midline (blue dashed line) at the level of the posterior margin of the eyelid (blue arrow). (**C)** Insertion of 2 skull-screw electrodes, one on each side of the skull (blue arrows). (**D**) Connection between the right electrode and a crocodile clip connected to the stimulator.

In sheep, the motor cortex is located in the superior frontal convolution of the medial part of the frontal lobe, *i.e*. between the cruciate sulcus posteriorly, the coronal sulcus laterally and the great longitudinal fissure medially. The hind- and the forelimb cortical motor representations are located in the posterior part of the superior frontal convolution. The cruciate sulcus is located at the level of the posterior margin of the orbit, while the hind- and forelimb representations are located between the midline and two cm lateral to the midline at the level of the posterior margin of the eyelid^21,29^. Single channel electrode implantation locations were determined based on external skin and skull landmarks (Fig. 1A)^21,29^. One pair of stimulating stainless-steel skull-screw electrodes (4.7 mm length-1.17 mm shaft diameter, #19010-00, Fine Science Tools, GmbH, Heidelberg, Germany) was used per lamb. Two burr holes were drilled symmetrically into both sides of the skull overlying the hind- and forelimb motor cortex area, two cm lateral to the midline at the level of the posterior margin of the orbit (Fig. 1B–D). Half of the length of the electrode was screwed into the hole so that the electrode would remain in the skull yet outside the brain.

**Figure 2:**
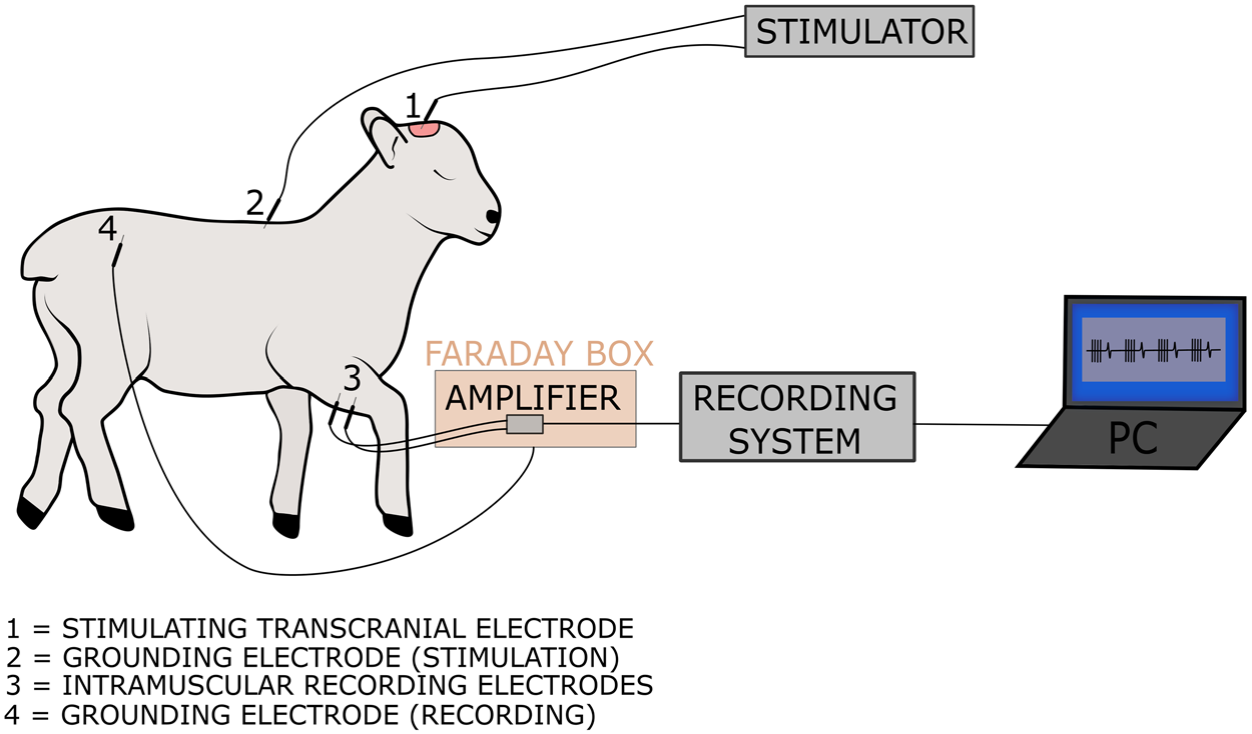
Schematic illustration of the experimental setup. The motor cortex was stimulated via transcranial screws using a constant-voltage stimulator. We used a wired recording setup to record MEPs in each limb. We stimulated both left and right hemisphere and recorded ipsi- and contralateral MEPs for both forelimbs and hindlimbs. Drawing by Marjolijn Deprez for and copyright by KU Leuven, Belgium.

One pair of intra-muscular needle recording electrodes (SDN electrodes RD/BK 12/2000, stainless steel; inomed Medizintechnik GmbH, Emmendingen, Germany) were inserted bilaterally in the peroneus longus muscle of the hindlimb and in the extensor carpis ulnaris muscle of the forelimb, respectively, 10–15 mm apart (Fig. 2).

### Feasibility study of MEP recording

Our MEP protocol followed the guidelines of both the American Society of Neurophysiological Monitoring and the American Clinical Neurophysiology Society^2,5^. Transcranial electrical stimulation was delivered with a constant-voltage stimulus generator and isolation unit (DS8000 Digital Stimulator and DLS100 isolator, World Precision Instruments, Sarasota, FL, USA). A stimulus was defined as a train of four anode-first biphasic pulses defined as follows: frequency of 500 Hz, pulse width per phase of 1 ms, interphase gap of 0.5 ms. Electrical stimulation was applied between one skull-screw electrode located at the motor cortex and a reference needle electrode (18 G × 1½”, BD Microlance^TM^ 3, Becton, Dickinson and Company Limited, Drogheda, Ireland) located subcutaneously at the thoracocervical region (Fig. 2). Stimulation intensities between 0 and 60 V for the forelimbs, and 0 and 100 V for the hindlimbs were applied in order to trigger a contraction of the limbs. For each limb, four stimuli were triggered manually every 2–3 s.

**Figure 3:**
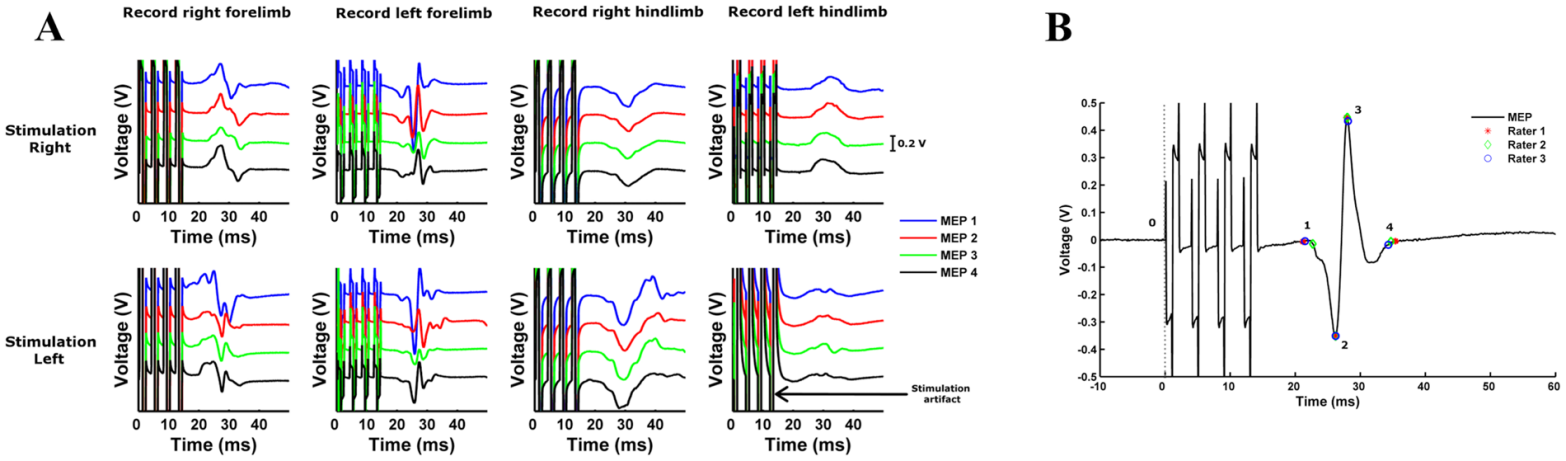
Visualization and analysis of MEPs with a custom-made MATLAB® algorithm. (**A)** Both ipsi- and contralateral MEPs were recorded for left and right motor cortex stimulation. Per limb, 4 stimuli were given and consequently 4 MEPs were recorded. Stimulation intensities ranged between 0–60 V for the forelimbs and 0–100 V for the hindlimbs. Based on the guidelines, the motor threshold was defined as the lowest stimulus intensity of motor cortex stimulation required to elicit at least 3 MEPs of similar shape^5^. (**B)** At motor threshold, response onset (point 1), maximal and minimal peaks (point 2 and 3) and the end of the response (point 4) were defined by 3 independent and blinded observers. These measurements were then used to calculate latency, area-under-the-curve, peak-to-peak and duration of each MEP at the motor threshold.

A wired setup consisting of a preamplifier, portable-ME recording System and pc software was used (hardware: *in vivo* USB-ME32-FAI-System 32 channel; software: MC-Rack; both from Multi Channel systems MCS GmbH, Reutlingen, Germany). To prevent noise, the preamplifier was first wrapped in aluminum foil and then encased in a stainless steel ‘Faraday’ box which was connected to the lamb using a subcutaneous needle (18 G × 1½”) in the lumbar-sacral region. MEPs were recorded between two electrodes at the earlier described locations (Fig. 2). The stimuli were also recorded as triggers in a separate channel. One recording consisted of four stimuli and four MEPs (each stimulus was followed by one MEP) to ensure replicability of the response. For each hemisphere, we recorded responses in both ipsilateral and contralateral limbs (Fig. 3).

### Validity study

To validate the above standardized set up, we performed two additional experiments. First, to confirm the presence of genuine MEPs, we administered intravenous curare in one lamb to elicit total NMB impeding muscle contraction hence eliminating the myogenic MEP^5^. Curare (vecuronium bromide 0.1 mg/kg, Norcuron 4 mg/mL, NV Organon-MSD, Oss, the Nederlands) was injected in one lamb under endotracheal intubation. Another lamb underwent transection of the spinal cord in the middle part of the lumbar region between spinal levels L3 and L4. For both experiments, MEPs were recorded in hindlimbs before and after each procedure.

### Quantitative MEP analysis

We applied a standardized analysis method using a custom-made algorithm in MATLAB® (Mathworks, Natick, MA, USA). Data were filtered between 30 and 1500 Hz using a second order Butterworth filter^2^. The timing of stimulation onset was detected using the data from the trigger channel. A 50 ms time window was chosen and defined as the time between first pulse of the stimulus and 50 ms afterwards^2^. To facilitate and standardize the analysis, a Graphical User Interface (GUI) was designed.

For each lamb, the analysis was performed in two steps. First, the responses to a specific stimulation position (left or right cortex) were displayed in one window with multiple panels corresponding to different amplitudes of stimulation (Fig. 3). For each stimulation amplitude, the responses to the four stimuli were displayed above each other to facilitate the analysis. The observer then selected the lowest stimulus intensity that elicited at least three MEPs of similar shape (*i.e*. the motor threshold) by clicking on the corresponding window. Second, responses were displayed in separate windows and the observer selected response onset (*i.e*. the lowest point before the MEP starts after repolarization from stimulus), maximal and minimal peaks and the end of the response (*i.e*. the beginning of a plateau of the resting phase). Whenever one stimulus evoked multiple MEPs, point four was defined as the intersection between the zero axis and the end of the first MEP (denoted by 1, 2, 3 and 4 on Fig. 3B). Point 0 was automatically denoted by the algorithm. Note that time was not displayed on the x-axis to prevent bias in choosing MEP onset.

From these four points, the software computed four measurements: latency in milliseconds (from 0 to 1), area-under-the-curve (AUC; from point 1 to 4), peak-to-peak amplitude in volts (P2P; measure from point 2 to 3) and duration in milliseconds (from 1 to 4). AUC was computed as the integral of the absolute value of the response between point 1 and point 4. These four parameters alongside motor threshold were chosen to quantitatively analyze MEPs in neonatal lambs^5^. This was based on previous pilot clinical studies showing the interest of these parameters to assess corticospinal excitability^22,30^. Motor threshold reflects the membrane excitability of the neurons in the cortical region of the target muscle^31^. Changes in MEP latency and duration may reflect variation in central motor conduction time^32,33^. The AUC provides a global estimate of the corticospinal excitability and its decrease indicates a decrease of excitability^34^ and P2P amplitude indirectly reflects the overall corticospinal response of the cortical neurons evoked by the stimulation^35^.

The analysis was repeated for each combination of stimulation position (left or right hemisphere) and limb recording (contra- or ipsilateral fore- or hindlimb)^5^. Analysis of the myogenic MEP raw data was performed by three independent who were blinded to origin of the recording. Therefore, the motor threshold was chosen by consensus by the three observers beforehand.

### Reliability analysis

Reliability is the ability to repeat or consistently obtain the same measurements under identical conditions^36^. Intra- and inter-observer reliabilities are respectively the ability of the same observer or different observers to obtain similar measurements consistently under the same circumstances. We analyzed reliability using the intraclass correlation coefficient (ICC) and the reliability coefficient (Cronbach’s α) for the 4 aforementioned biometric measurements (latency, AUC, P2P, duration)^36,37^. The intra-ICC estimates the overall correlation between all possible measurements within the variable taken by the same observer and inter-ICC for measurements by different observers. According to Kline, a value of α ≥ 0.7 is considered a reliable consensus for the intra-ICC and inter-ICC in the case of ability tests^38^. Statistical analysis was performed utilizing the Statistical Package for the Social Sciences (IBM SPSS® statistics, version 21, IBM Corporation, Amonk, NY, USA).

Bland-Altman analysis was used to assess the agreement between repeated measurements performed by one observer and between measurements performed by two observers^39,40^. We used Microsoft® Excel software (version 15.28, Microsoft® Corporation) to create these plots. To obtain a good agreement, the mean difference (bias) should be close to zero and 95% of the observations should be located within two standard deviations of the mean (±2 SD; limits of agreements)^39,40^.

### Data Availability

The datasets generated during and/or analyzed during the current study are available from the corresponding author on reasonable request.

## Results

### Feasibility and validity

The mean time interval between delivery and MEP recording was 36 ± 9.3 hours. The general health status as well as motor and sensory functions of all lambs were normal prior to MEP recording. Using the standardized MEP protocol developed during our pilot study (data not shown), we were able to record contra- and ipsilateral myogenic MEPs for the four limbs in all six neonatal lambs (mean weight, 3677 ± 179 grams).

**Figure 4:**
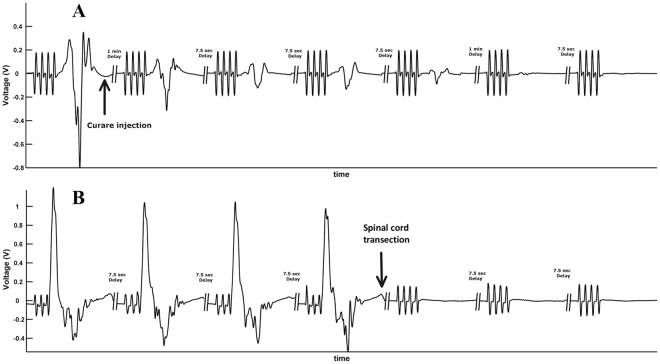
Validity analysis. (**A)** First, to confirm the presence of genuine MEPs, total neuro-muscular blockade was performed to eliminate myogenic MEP. One normal lamb was administered intravenous curare under orotracheal ventilation. Consequently, MEPs were recorded in all limbs before administration of curare and disappeared afterwards. (**B)** To confirm the applicability of our methodology in an animal model of spinal cord injury, another lamb underwent transection of the spinal cord. Similarly, MEPs were recorded in hindlimbs before transection of the spinal cord and disappeared afterwards.

Total NMB eliminated myogenic MEPs in the four limbs (Fig. 4A). Transection of the lumbar spinal cord under general anesthesia eliminated MEPs of the hindlimbs (Fig. 4B). In brief, these experiments demonstrate that in lambs, (i) MEPs can be recorded in the neonatal lamb, and (ii) can be used to assess motor function in spinal cord injury.

### Quantitative analysis and reliability

**Figure 5:**
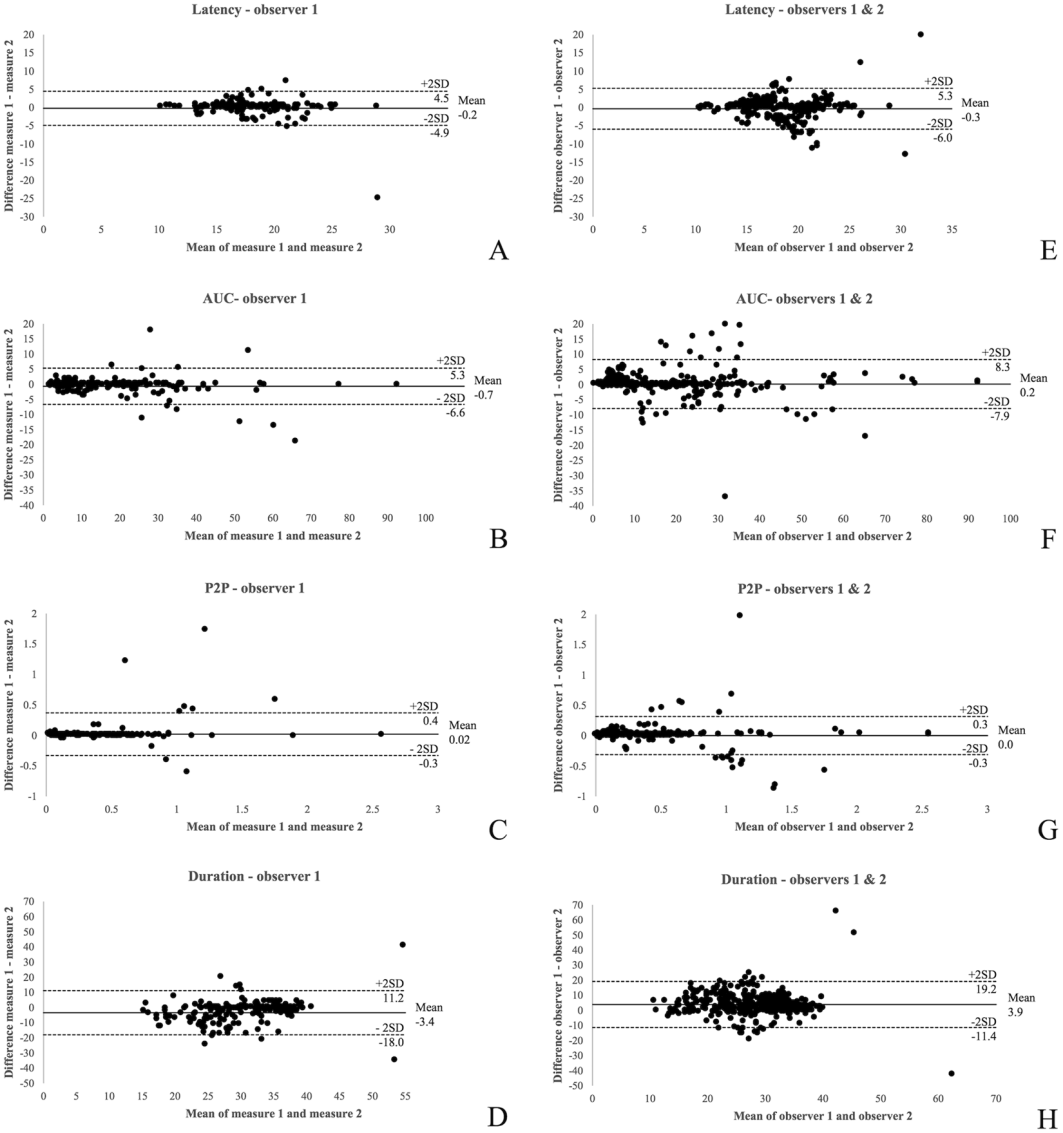
Bland-Altman plots for the 4 parameters taken by the same (observer 1; **A**–**D**) and different observers (observers 1 and 2; **E**–**H**). 4 parameters: latency in ms, area-under-the-curve (AUC), peak-to-peak (P2P) in Volts and duration in ms. ICC stands for intra-class correlation.

Table 1 shows an overview of the intra-ICC, inter-ICC and reliability alpha coefficients for the four biometric parameters. The coefficients were ≥ 0.7 for all parameters, demonstrating the reliability of the analysis method. However intra- and inter-observer Bland-Altman plots did not demonstrate any bias, except for duration. The latter had a high bias (−3.4 and 3.9 ms) and wide limits of agreement with an average duration of 30.4 ms (Fig. 5).

**Table 1:** Reliability analysis: intraobserver and interobserver intraclass correlation coefficients with 95% confidence intervals and reliability coefficient for the 4 biometric parameters in normal neonatal lambs.

**Parameter**	**intra-ICC (95% CI)**	**reliability coefficient (Cronbach’s α)**	**inter-ICC (95% CI)**	**reliability coefficient (Cronbach’s α)**
Latency	0.88 (0.856–0.904)	0.88	0.92 (0.895–0.938)	0.92
AUC	0.98 (0.977–0.985)	0.98	0.99 (0.991–0.994)	0.99
P2P	0.97 (0.962–0.975)	0.97	0.98 (0.976–0.985)	0.98
Duration	0.71 (0.637–0.772)	0.73	0.71 (0.626–0.774)	0.72

AUC, area-under-the-curve; P2P, peak-to-peak; ICC, intra-class correlation; CI, confidence intervals.

Table 2 displays the MEP data obtained from healthy control neonatal lambs of the motor threshold, latency, AUC, P2P and duration per limb. Again, these were comparable for contra- and ipsilateral MEPs in fore- and hindlimbs after stimulation of the same cortex.

**Table 2:** Descriptive statistics: physiologic MEP data obtained from healthy neonatal lambs for each of the 5 parameters per recording (threshold, latency, area-under-the-curve, peak-to-peak and duration). MEP, motor evoked potential; AUC, area-under-the-curve; P2P, peak-to-peak; R; right side; L, left side; FL, forelimb MEP recording; HL, hindlimb MEP recording; SD, standard deviation; ms, milliseconds.

MEP recording		Parameter	Mean ± SD	Range
Right motor cortex stimulation	R-FL	Threshold (Volts)	15.0 ± 5.80	10.0–25.0
		Latency (ms)	15.6 ± 2.38	10.2–23.2
		AUC	31.0 ± 20.78	8.1–92.6
		P2P (Volts)	0.68 ± 0.54	0.14–2.57
		Duration (ms)	30.9 ± 5.21	11.2–40.2
	L-FL	Threshold	18.4 ± 12.30	10.0–45.0
		Latency	16.9 ± 2.45	10.8–20.6
		AUC	16.3 ± 13.21	3.6–57.0
		P2P	0.48 ± 0.43	0.06–2.09
		Duration	25.8 ± 9.59	8.5–75.2
	R-HL	Threshold	26.7 ± 11.08	15.0–45.0
		Latency	20.7 ± 4.63	12.4–37.1
		AUC	7.1 ± 3.47	1.9–17.1
		P2P	0.19 ± 0.14	0.05–0.52
		Duration	25.0 ± 8.39	7.4–40.5
	L-HL	Threshold	30.0 ± 12.64	15.0–55.0
		Latency	19.8 ± 3.28	11.7–25.6
		AUC	12.6 ± 10.41	0.3–35.8
		P2P	0.23 ± 0.20	0.00–0.75
		Duration	27.3 ± 8.11	10.2–40.8
Left motor cortex stimulation	L-FL	Threshold	16.7 ± 4.73	10.0–25.0
		Latency	16.8 ± 1.57	13.8–21.5
		AUC	23.5 ± 11.03	5.1–59.4
		P2P	0.58 ± 0.45	0.01–2.06
		Duration	28.9 ± 8.62	3.0–83.6
	R-FL	Threshold	18.5 ± 3.92	15.0–25.0
		Latency	16.0 ± 3.57	12.9–44.2
		AUC	31.1 ± 15.00	1.3–75.6
		P2P	0.56 ± 0.24	0.21–1.39
		Duration	31.0 ± 5.76	8.7–41.1
	L-HL	Threshold	24.2 ± 8.41	15.0–35.0
		Latency	19.4 ± 2.70	14.7–24.7
		AUC	10.3 ± 6.19	2.5–22.6
		P2P	0.20 ± 0.12	0.05–0.44
		Duration	28.1 ± 7.70	12.8–39.9
	R-H	Threshold	31.7 ± 12.18	20.0–55.0
		Latency	19.8 ± 3.76	14.6–41.7
		AUC	8.6 ± 8.04	1.2–36.4
		P2P	0.17 ± 0.14	0.02–0.64
		Duration	26.4 ± 8.88	9.3–71.4

## Discussion

We demonstrated the feasibility and reliability of this standardized custom-made algorithm, using three parameters (*i.e*. latency, AUC and P2P amplitude), for myogenic MEP recordings in neonatal lambs. We left duration out of the analytic method because of high bias. Validity was demonstrated by pharmacologic and surgical NMB. Though the recordings may remain time consuming, we think time can be saved during the analysis.

MEPs test electrical conduction within the upper and lower motor neurons of the corticospinal pathways. Due to their topography and vascularization, these motor pathways are more sensitive to ischemic insults than somatosensory pathways^1,5^. As a result, MEPs can be used to specifically assess the functional integrity of the spinal cord, nerve roots and peripheral nerves and are more valuable to evaluate motor performance compared to other recording methods such as SEPs^5^. MEP recordings have been used to evaluate motor function in different animal models of brain, spinal cord and nerve injuries or diseases, including mice, rats, rabbits and adult sheep^6,11–15,41^. However, the interpretation of MEPs has been limited by qualitative or quantitative methods used, the latter relying only on latency and amplitude. We have introduced additional quantitative measures such as motor threshold, AUC, P2P amplitude and duration, which were earlier proposed to be interesting to assess corticospinal excitability^22,30^. The herein described method allows researchers to analyze their MEP recordings comprehensively and reliably using three of these parameters, *i.e*. latency, AUC and P2P amplitude. The relevance of these three parameters is supported by their ability to assess motor corticospinal function, respectively estimating central motor conduction time^32,33^, corticospinal excitability^34^ and corticospinal response of the evoked cortical neurons^35^.

This method, however, may not be limited to experimental animals. Clinically, during or after IONM, MEP interpretation is also subjective, and based on binary and semi-quantitative criteria such as MEP disappearance, amplitude reduction, acute threshold elevation or morphology simplification^2,5^. It is possible to use the herein studied standardized analysis method both on-line or off-line, and could be programmed to also detect quantitative MEP alarm- or cut off criteria, as recently suggested by Segura *et al*. for the early detection motor function impairment or recovery^5,22^.

For clinical MEP recordings, subcutaneous cork-screw electrodes are typically used. These electrodes stimulate the brain through the skull, and therefore require high stimulation intensities (up to 900 V) to elicit MEPs^2^. Such a setting has also been used in adult sheep^13^. Since the maximal voltage that our animal stimulator could apply was 120 V, we could not use cork-screw electrodes. We rather implanted skull-screw electrodes partially into the skull and eventually never required more than 100 V. Unlike in humans, MEPs did not reappear after curarization, even after waiting for three hours. This could be due to the duration of the curarization by vecuronium as well as the age of the animal. Despite its short half-life of 36 to 75 minutes, infants under the age of one, are more sensitive to vecuronium than adults. Therefore, it takes about 50% longer to recover than in adults^42–44^.

Apart from the MEP recordings, we gathered physiologic MEP data obtained from healthy control neonatal lambs. We could not directly confront these to findings in the same animal model yet in other studies. To the best of our knowledge, transcranial myogenic MEPs have never been recorded in neonatal lambs. Non-myogenic MEPs were earlier reported in adult sheep, i.e. epidural recordings after cortical stimulation and peripheral nerve, recordings after transcranial stimulation or muscle recordings after spinal nerve root stimulation^13,15,45^.

Intra-individual variability has been reported, yet it is usually explained as due to varying fractions of horn cells and lower motor neurons pools being activated with each consecutive stimulus^5,46^. In our study however, we observed a low intra-individual variability in MEP response in our lambs. This is in concordance with the single previous study on MEP in adult sheep^13^. Similar to previous reports in adult sheep^11,46,47^, we observed large inter-individual variability^13,15,45^. Many factors such as the sex of the animal, cable and device quality, level of anesthesia, core temperature and nutritional status of the animal have been named to explain this variability^5,47^. A number of factors out of this list were present in our experimental setting. This mainly concerned sex as, due to ethical considerations, we did not restrict our experiments to lambs of one sex. Other factors included fluctuations in the level of anesthesia, nutritional status or body temperature.

In brief, we developed a standardized methodology to record and quantitatively analyze MEPs in neonatal lambs, as part of our ongoing research on the efficacy of prenatal spina bifida repair techniques^16,48^. This analysis method may be used more widely in other animal models of spinal cord or brain injury, potentially also clinically.
